# The development and validation of a predictive model for neonatal phototherapy outcome using admission indicators

**DOI:** 10.3389/fped.2022.745423

**Published:** 2022-10-11

**Authors:** Qin Liu, Zaixiang Tang, Huijun Li, Yongfu Li, Qiuyan Tian, Zuming Yang, Po Miao, Xiaofeng Yang, Mei Li, Lixiao Xu, Xing Feng, Xin Ding

**Affiliations:** ^1^ Soochow Key Laboratory of Prevention and Treatment of Child Brain Injury, Children's Hospital of Soochow University, Suzhou, China; ^2^ Department of Neonatology, Suzhou Science / Technology Town Hospital, Suzhou, China; ^3^ Department of Biostatistics, School of Public Health, Medical College of Soochow University, Suzhou, China; ^4^ Institute of Pediatric Research, Children’s Hospital of Soochow University, Suzhou, China; ^5^ Neonatology Department, The Affiliated Suzhou Hospital of Nanjing Medical University, Suzhou Municipal Hospital, Suzhou, China

**Keywords:** bilirubin encephalopathy, complication, exchange transfusion therapy, phototherapy failure, predictive model, admission indicator, neonatal hyperbilirubinemia

## Abstract

Delayed exchange transfusion therapy (ETT) after phototherapy failure for newborns with severe hyperbilirubinemia could lead to serious complications such as bilirubin encephalopathy (BE). In this current manuscript we developed and validated a model using admission data for early prediction of phototherapy failure. We retrospectively examined the medical records of 292 newborns with severe hyperbilirubinemia as the training cohort and another 52 neonates as the validation cohort. Logistic regression modeling was employed to create a predictive model with seven significant admission indicators, i.e., age, past medical history, presence of hemolysis, hemoglobin, neutrophil proportion, albumin (ALB), and total serum bilirubin (TSB). To validate the model, two other models with conventional indicators were created, one incorporating the admission indicators and phototherapy failure outcome and the other using TSB decrease after phototherapy failure as a variable and phototherapy outcome as an outcome indicator. The area under the curve (AUC) of the predictive model was 0.958 [95% confidence interval (CI): 0.924–0.993] and 0.961 (95% CI: 0.914–1.000) in the training and validation cohorts, respectively. Compared with the conventional models, the new model had better predictive power and greater value for clinical decision-making by providing a possibly earlier and more accurate prediction of phototherapy failure. More rapid clinical decision-making and interventions may potentially minimize occurrence of serious complications of severe neonatal hyperbilirubinemia.

## Introduction

Jaundice is a common neonatal condition found in 60% of term and 80% of preterm infants in the first week after birth (1). Excessive bilirubin in the circulation could cross the newborn’s immature blood brain barrier (BBB) and damage the central nervous system. This could result in acute bilirubin encephalopathy (ABE) and irreversible consequences such as visual and auditory impairments and cerebral palsy (2). Risks for serious complications are even greater in newborns with severe hyperbilirubinemia (3). Three out of 14 infants with evident bilirubin encephalopathy (BE) die as reported in a British prospective study (4). 1–3 newborns per 100,000 live births are at increased risks for kernicterus if their hyperbilirubinemia is not treated and 5%–10% of the survivors may suffer permanent complications (5). Clinical interventions including phototherapy, medications, and exchange transfusion therapy (ETT) are used to reduce highly neurotoxic bilirubin levels, among which phototherapy remains the safest, most common, and effective treatment (6). However, a variety of factors ranging from preterm delivery, infections, to an underdeveloped liver enzyme system, intestinal hepatic circulation, and BBB, may decrease the efficacy or even cause failure of phototherapy (7–9). If phototherapy is ineffective, ETT within 2 weeks after birth may be performed, even a second transfusion in some cases. Hyperbilirubinemia may even progress into ABE (10), a preventable outcome if phototherapy failure could be identified early.

The decision whether an ETT is needed is often made predicated on the condition of the neonate, including presence of hemolysis, signs of BE, and efficacy of phototherapy. The definition of phototherapy failure is a TSB level >8.6 μmol/(L·h) [0.5 mg/(dl·h)] 4–6 h after phototherapy is initiated (11). This, however, considers only changes in TSB and fails to integrate other factors such as clinical presentation and laboratory findings on admission. As a result, a predictive instrument using clinical data on admission may allow earlier prediction of phototherapy outcome and guide clinical decision-making.

Efforts to develop predictive models have largely been focused on risk factors for hyperbilirubinemia and ABE instead of phototherapy outcome. A risk factor scoring system was created to predict occurrence of hyperbilirubinemia and an associated increase in risk with heavier birth weight (12). Chang et al. developed a clinical prediction model for ABE using the bilirubin/albumin (B/A) ratio, risk factors for hyperbilirubinemia, and amplitude-integrated electroencephalography (aEEG) data. The model showed reasonable predicting power and was believed to improve accuracy of diagnosing ABE (13) but lacked specific evaluation indicators and failed to yield adequate evidence for assessing probability of ABE based solely on aEEG data. Bhutani et al. created the bilirubin-induced-neurologic dysfunction (BIND) score for early identification of BE (14). Yet the development of bilirubin neurotoxicity is complex. A very high TSB level does not always lead to kernicterus while some other risk factors could amplify bilirubin toxicity. Kernicterus has even been reported in cases of physiological or breast milk jaundice (15), which could eventually result in ABE if ETT is delayed by ineffective phototherapy. Early prediction of phototherapy outcome, therefore, is imperative for improving clinical decision-making. However, we have found no previous literature reporting an accurate predictive model for neonatal phototherapy outcomes using admission data. The current study aimed to develop and validate such a model with acceptable accuracy. The new model could be used to reduce the severe complications of severe neonatal hyperbilirubinemia.

## Materials and methods

### Patients

In creating and validating the predictive model, we retrospectively examined the medical records of newborns with hyperbilirubinemia from two tertiary hospitals in East China to build two cohorts, a training cohort for model development and a validation cohort for model testing. The inclusion criteria for subject selection were as follows: (a) gestational age ≥35 weeks; (b) age ≤28 days; and (c) a TSB level ≥205.2 μmol/L (12 mg/dl) 4–6 h after phototherapy if patient age ≤24 h, TSB ≥290.7 μmol/L (17 mg/dl) if age >24 h and ≤48 h, TSB ≥342. 0 μmol/L (20 mg/dl) if age >48 and ≤72 h, or TSB ≥376.2 μmol/L (22 mg/dl) if age >72 h (16). Exclusion criteria were: (a) presence of unstable internal environment, intracranial hemorrhage, congenital craniocerebral malformation, congenital gastrointestinal malformation, or congenital epilepsy in the newborn; (b) history of epilepsy in immediate family members; (c) mother taking psychiatric medications before childbirth; (d) evidence of serious inherited metabolic diseases; (e) BE manifestations on admission, such as increased muscle tone, body flexion, head tilt, opisthotonus, fever, and high-pitched cry; (f) self-discharge within 24 h of hospitalization; and (g) missing significant clinical data.

#### Training and validation cohorts

Newborns diagnosed with severe neonatal hyperbilirubinemia (*N* = 292) admitted to the Neonatal Intensive Care Unit (NICU), Children's Hospital of Soochow University, a tertiary medical center in East China, between 1 January 2017 and 30 April 2021 were included in the training cohort for building a predictive model. Newborns with severe hyperbilirubinemia (*N* = 52) admitted to the NICU of Children’s Hospital of Soochow University and Suzhou Municipal Hospital, another tertiary hospital in the same city, between 1 May 2021 and 30 June 2021 were included in the validation cohort.

#### Patient data

All included neonates received phototherapy. The following data were retrieved from the hospitals’ electronic medical record systems: general data (gender, age, birth weight, gestational age, type of delivery, Apgar score), past medical history [unexplained stillbirth, miscarriage, BE, glucose-6-phosphate dehydrogenase (G6PD) deficiency, intrauterine hemolysis], clinical diagnosis, hemolytic jaundice (ABO/Rh hemolysis, G6PD deficiency), routine blood test results [white blood cell (WBC), neutrophil proportion (N%), hemoglobin (Hb)], reticulocyte ratio and count, potential of hydrogen (pH), blood biochemistry [TSB, albumin (ALB), C-reactive protein (CRP)], clinical manifestations (screaming, muscular tone, lethargy, reaction, sucking), ALB and gamma globulin therapies, ETT, and findings of magnetic resonance imaging (MRI) and brainstem auditory evoked potential (BAEP) examination. G6PD deficiency screening was not performed for all included newborns. Other significant clinical information such as TSB level after phototherapy and use of ETT and other interventions was also collected.

### Clinical protocol

All newborns were diagnosed with severe neonatal hyperbilirubinemia according to the Chinese clinical guidelines (17). Phototherapy was routinely ordered, following which TSB was tested after 4–6 h to determine efficacy. Newborns with hemolytic hyperbilirubinemia were routinely given intravenous immunogloblin (IVIG) and ALB therapies. Probiotics was administered as an auxiliary therapy if oral feeding was feasible.

#### Phototherapy

The newborns had their eyes covered with light-shielding goggles and perineum covered with a diaper, with the remaining skin exposed. They were placed in a supine position in an incubator and were radiated with double-sided blue light [wavelength 425–475 nm, intense light therapy 30 μW/(cm^2^·nm)] for 4–6 h. Body temperatures were closely monitored. Fluid supplementation was given during therapy. Auxiliary probiotics therapy was routinely used to block the enterohepatic circulation for the newborns on enteral feeding.

The TSB level was tested again after phototherapy if not otherwise indicated. A TSB test was ordered during phototherapy for the hemolytic newborns or those with a TSB level close to the threshold for ETT. Reassessments and interventions were made if signs of phototherapy failure were noted.

#### ETT

The protocol of ETT was as follows: selection of blood: (a) in case of Rh incompatibility, blood of the same Rh group as the mother, the same ABO group as the newborn, or Type O blood was selected. Rh+ blood was used in case of Rh (anti-D) hemolysis without available Rh- blood; (b) in case of ABO incompatibility, Type AB plasma mixed with Type O red blood cells was preferred for exchange transfusion. Type O or the same type as the infant was also acceptable; (c) for patients with evident severe heart failure, concentrated blood with halved plasma was used to correct anemia and heart failure; (d) fresh blood was preferred. Cryopreserved blood was used if fresh blood was not available. Blood was preheated at room temperature to approximate body temperature before transfusion; anticoagulant: 3–4 mg heparin was added per 100 ml blood. Protamine at half the heparin quantity was administered after transfusion for neutralization. Because citrate maintenance solution could bind with free calcium and cause hypocalcemia, 1 ml 10% calcium gluconate was slowly injected per 100 ml blood. Another 2–3 ml was slowly injected after transfusion; exchange transfusion: routes of transfusion: umbilical vein, umbilical artery and vein simultaneously, and peripheral blood vessels. Quantity of blood exchange: 150–180 ml/kg, 400–600 ml in total. The transfusion was administered at 3–5 ml/kg each time, at an even drip rate of about 10 ml/min. Phototherapy was continued after ETT. Heart rate and respiration were checked every 4 h. TSB level, lethargy, refusal to eat, irritability, convulsions, embrace reflex, and other signs were closely monitored. Antibiotic prophylaxis was administered for 3 days. Routine blood tests were performed every 1–3 days, TSB tested daily, red blood cell (RBC) and Hb measured every 2 weeks until 2 months after birth.

### Predictive model development and validation

Stepwise logistical regression modeling was employed to develop the predictive model (Model 1) using the admission data from the training cohort. Odds ratios (OR) and 95% confidence intervals (95% CI) were calculated for each indicator. Stepwise regression according to the Akaike Information Criteria (AIC) was performed to select the predictors from the training cohort. A nomogram was created for Model 1. Discrimination and calibration of the model were evaluated with AUCs and calibration plots.

To compare the predictive power of Model 1 and conventional predictors, two more models were built (Models 2 and 3). A linear predictive value was yielded with the predictors of Model 1. It was combined with the indicator of TSB decrease after phototherapy as an independent variable to create Model 2. Model 3 was created using TSB decrease after phototherapy as a variable and phototherapy outcome as an outcome indicator. Discrimination and calibration of the models were evaluated with AUCs and calibration plots.

**Figure 1:**
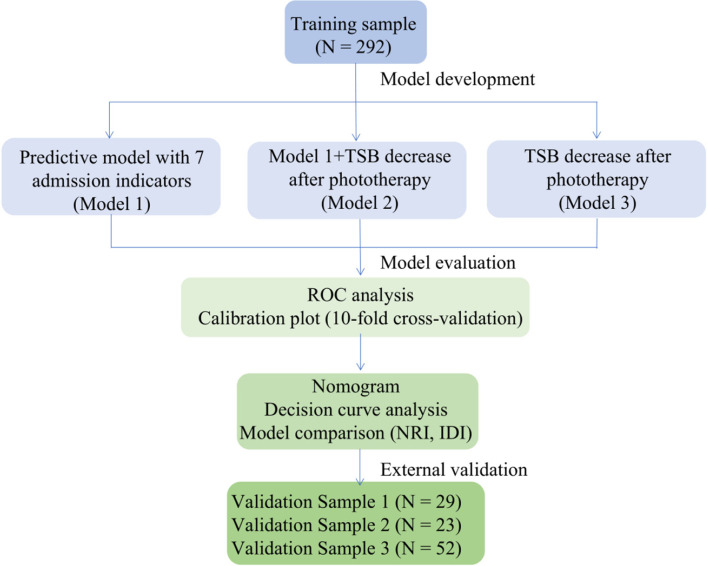
Workflow of predictive model development and validation. TSB, total serum bilirubin; ROC, receiver operator characteristics; NRI, net reclassification improvement; IDI, integrated discrimination improvement.

The workflow of model development and validation is in Figure 1.

#### Measures of validation

The three models were compared using AUCs, calibration plots, decision curve analyses (DCA), net reclassification improvements (NRI), and integrated discrimination improvements (IDI).

### Statistical analyses

We used the R software (version 4.1.0, https://www.r-project.org/) for statistical analyses. Quantitative data were expressed as medians and interquartile ranges. Shapiro–Wilk test was used to test normality, *t*-test for intergroup comparisons of normally distributed data, and Wilcoxon’s rank sum test for intergroup comparisons of non-normally distributed data. Qualitative data were expressed as frequencies and constituent ratios. Intergroup comparisons of qualitative data were made with *Chi*-square test. *p* < 0.05 was statistically significant.

### Ethical consideration and informed consent

The study was ethically approved by the Central Ethics Committee of the Children's Hospital of Soochow University (number 2016028). Informed consents were signed by the legal guardians of the newborns.

## Results

### Characteristics of subjects

**Figure 2:**
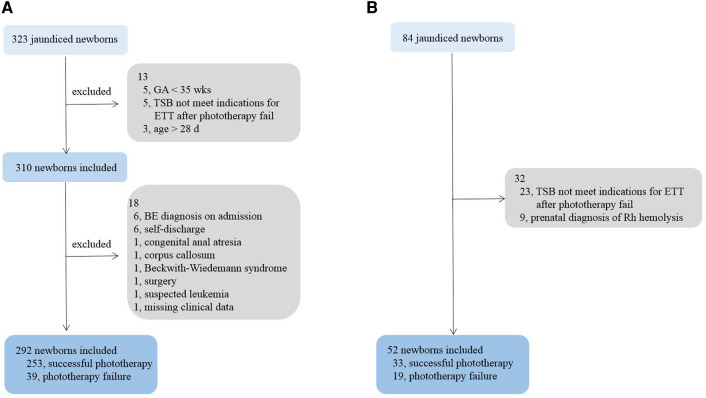
Process of study subject selection. (**A**) training cohort; (**B**) validation cohort. GA, gestational age; TSB, total serum bilirubin; BE, bilirubin encephalopathy.

For the training cohort, we enrolled a total of 323 neonates, who received phototherapy. Preliminary screening excluded five neonates due to gestational ages <35 weeks, five due to TSB levels not meeting the threshold for ETT after phototherapy failure, and three for age >28 days. Further screening excluded another six cases with a BE diagnosis on admission, six for self-discharge within 24 h after hospitalization, one for congenital anal atresia, one for orpus callosum tumor, one for suspected Beckwith–Wiedemann syndrome, one undergoing a surgery, one for suspected leukemia, and one for missing significant clinical data. We eventually included 292 newborns in the training cohort, including 170 males (58%). Figure 2 displays the process of study subject selection.

There were significant differences in age, past medical history, hemolysis, Hb, N%, ALB, TSB, and TSB decrease after phototherapy between the groups with successful and failed phototherapy. Table 1 details the demographic and clinical characteristics of the newborns in the training cohort.

**Table 1:** Demographic and clinical characteristics of newborns in training cohort (*N* = 292).

Characteristic	Total (*N* = 292)	Successful phototherapy group (*n* = 253)	Phototherapy failure group (*n* = 39)	*p* value
Gender				0.674
Male	170 (58%)	149 (59%)	21 (54%)	
Female	122 (42%)	104 (41%)	18 (46%)	
Age, day	7.00 (5.00, 9.00)	7.00 (5.00, 10.00)	4.00 (1.55, 6.00)	<0.001
GA, week	38.86 (38.00, 39.86)	38.86 (38.00, 39.71)	39.00 (38.00, 40.00)	0.633
Birth weight, gram				0.609
<2,500	1 (0.3%)	1 (0.4%)	0 (0%)	
2,500–4,000	266 (91%)	229 (91%)	37 (95%)	
>4,000	25 (8.6%)	23 (9.1%)	2 (5.1%)	
Past medical history				<0.001
No	276 (95%)	245 (97%)	31 (79%)	
Yes	16 (5.5%)	8 (3.2%)	8 (21%)	
Hemolysis				<0.001
No	250 (86%)	231 (91%)	19 (49%)	
Yes	42 (14%)	22 (8.7%)	20 (51%)	
WBC, 10^9^/L, median (IQR)	10.33 (8.82, 12.12)	10.21 (8.64, 11.76)	11.47 (9.93, 16.67)	<0.001
Hb, g/L, median (IQR)	169 (153, 187)	171 (159, 189)	144 (126, 162)	<0.001
N%, median (IQR)	38 (30, 48)	37 (29, 44)	60 (46, 67)	<0.001
ALB, g/L, median (IQR)	38.10 (36.40, 39.92)	38.20 (36.60, 40.00)	35.40 (30.80, 38.55)	<0.001
TSB, mmol/L, median (IQR)	425 (406, 459)	423 (406, 450)	492 (409, 541)	0.002
TSB decrease after phototherapy				<0.001
>2–3 mg/dl	224 (77%)	218 (86%)	6 (15%)	
<2–3 mg/dl	68 (23%)	35 (14%)	33 (85%)	

GA, gestational age; WBC, white blood cell; IQR, interquartile range; Hb, hemoglobin; N%, neutrophil proportion; ALB, albumin; TSB, total serum bilirubin.

A total of 33 candidate newborns were selected for the validation cohort from Children’s Hospital of Soochow University. Subsequent screening excluded two cases for prenatal diagnoses of Rh hemolysis, neither with indications for ETT after birth, and two cases for a TSB level not meeting the threshold for ETT after phototherapy failure. Eventually, 29 newborns were included in the validation cohort. Table 2 details their demographic and clinical characteristics.

**Table 2:** Demographic and clinical characteristics of newborns in validation cohort: Children’s Hospital of Soochow University (*N* = 29).

Characteristic	Total (*N* = 29)	Successful phototherapy group (*n* = 26)	Phototherapy failure group (*n* = 3)	*p* value
Age, day	7.00 (5.00, 9.00)	7.50 (5.00, 9.80)	5.00 (2.65, 6.00)	0.222
Past medical history				0.002
Yes	2 (6.9%)	0 (0%)	2 (67%)	
No	27 (93%)	26 (100%)	1 (33%)	
Hemolysis				0.1127
Yes	5 (17%)	3 (12%)	2 (67%)	
No	24 (83%)	23 (88%)	1 (33%)	
Hb, g/L, median (IQR)	14 (8, 19)	15 (9, 20)	5 (3, 6)	0.002
N%, median (IQR)	39 (33, 46)	38 (31, 48)	42 (40, 42)	0.517
ALB, g/L, median (IQR)	39.40 (38.30, 40.90)	40.10 (38.83, 41.12)	35.40 (31.20, 36.55)	0.016
TSB, mmol/L, median (IQR)	15 (8, 22)	14 (8, 21)	25 (13, 27)	0.714
Decrease				
>2–3 mg/dl	25 (86%)	24 (92%)	1 (33%)	0.0548
<2–3 mg/dl	4 (14%)	2 (7.7%)	2 (67%)	

Hb, hemoglobin; IQR, interquartile range; N%, neutrophil proportion; ALB, albumin; TSB, total serum bilirubin.

Another 23 eligible newborns were selected from Suzhou Municipal Hospital. See Table 3 for their demographic and clinical characteristics.

**Table 3:** Demographic and clinical characteristics of newborns in validation cohort: Suzhou Municipal Hospital (*N* = 23).

Characteristic	Total (*N* = 23)	Successful phototherapy group (*n* = 7)	Phototherapy failure group (*n* = 16)	*p* value
Age, day	6.50 (1.71, 9.27)	8.00 (6.13, 9.50)	6.25 (1.25, 8.39)	0.216
GA, week	39.29 (38.00, 40.28)	38.71 (38.00, 40.64)	39.43 (38.03, 40.14)	0.987
Birth weight, gram				1.000
<2,500	1 (4.3%)	0 (0%)	1 (6.2%)	
2,500–4,000	22 (96%)	7 (100%)	15 (94%)	
Past medical history				1.000
No	22 (96%)	7 (100%)	15 (94%)	
Yes	1 (4.3%)	0 (0%)	1 (6.2%)	
Hemolysis				0.094
No	12 (52%)	6 (86%)	6 (38%)	
Yes	11 (48%)	1 (14%)	10 (62%)	
Hb, g/L, median (IQR)	152 (134, 178)	152 (141, 189)	152 (133, 175)	0.427
N%, median (IQR)	50 (32, 66)	33 (29, 38)	65 (44, 69)	0.019
ALB, g/L, median (IQR)	35.00 (32.75, 37.60)	32.90 (31.70, 35.45)	36.30 (33.05, 38.17)	0.141
TSB, mmol/L, median (IQR)	423 (373, 608)	394 (373, 427)	504 (374, 618)	0.171
TSB decrease after phototherapy				0.198
>2–3 mg/dl	17 (74%)	7 (100%)	10 (62%)	
<2–3 mg/dl	6 (26%)	0 (0%)	6 (38%)	

GA, gestational age; Hb, hemoglobin; IQR, interquartile range; N%, neutrophil proportion; ALB, albumin; TSB, total serum bilirubin.

### Model development

#### Model 1

The training cohort data were analyzed with logistical regression. Seven admission indicators were found to be significantly different between the successful phototherapy group and the phototherapy failure group (*p* < 0.01), including age, past medical history, hemolysis, Hb, N%, ALB, and TSB. Difference in TSB decrease after phototherapy was also statistically significant (*p* = 0.002). A predictive model (Model 1) was then created using stepwise logistic regression analysis of the seven significant admission indicators (Table 4).

**Table 4:** Model 1 created with stepwise logistic regression analysis of seven admission indicators.

Indicator	Coefficient	Standard error	OR (95% CI)	*p* value*
	0.496	3.891	—	0.899
Age	−0.414	0.137	0.661 (0.506–0.864)	0.002
Past medical history	2.234	0.829	9.337 (1.839–47.410)	0.007
Hemolysis	1.603	0.645	4.968 (1.403–17.588)	0.013
Hb	−0.033	0.010	0.968 (0.949–0.987)	0.002
N%	0.036	0.025	1.037 (0.987–1.089)	0.150
ALB	−0.17	0.08	0.84 (0.72–0.99)	0.033
TSB	0.021	0.005	1.021 (1.011–1.031)	<0.001

OR (95% CI), odds ratio (95% confidence interval); Hb, hemoglobin; N%, neutrophil proportion; ALB, albumin; TSB, total serum bilirubin.

* *p* < 0.05 was statistically significant.

#### Model 2

Two additional models were created for validating Model 1. A linear predictive value was generated with the predictors of Model 1 and combined as a variable with TSB decrease after phototherapy to create Model 2 (Table 5).

**Table 5:** Model 2 created with linear predictive value of Model 1 and TSB decrease after phototherapy.

Characteristic	Coefficient	Standard error	OR (95% CI)	*p* value*
	0.923	0.396	—	0.020
Model 1	0.766	0.138	2.151 (1.641–2.819)	<0.001
TSB decrease after phototherapy	2.680	0.663	14.588 (3.978–53.000)	<0.001

OR (95% CI), odds ratio (95% confidence interval); TSB, total serum bilirubin.

* *p* < 0.05 was statistically significant.

#### Model 3

Model 3 was a logistic regression model. It was created using TSB decrease after phototherapy as the only variable and the phototherapy outcome as the outcome indicator (Table 6).

**Table 6:** Model 3 created with TSB decrease after phototherapy and phototherapy outcome.

Characteristic	Coefficient	Standard error	OR (95% CI)	*p* value*
	−0.059	0.243	—	0.808
TSB decrease after phototherapy	−3.534	0.480	0.029 (0.011–0.075)	<0.001

OR (95% CI), odds ratio (95% confidence interval).

* *p* < 0.05 was statistically significant.

### Predictive model validation

**Figure 3:**
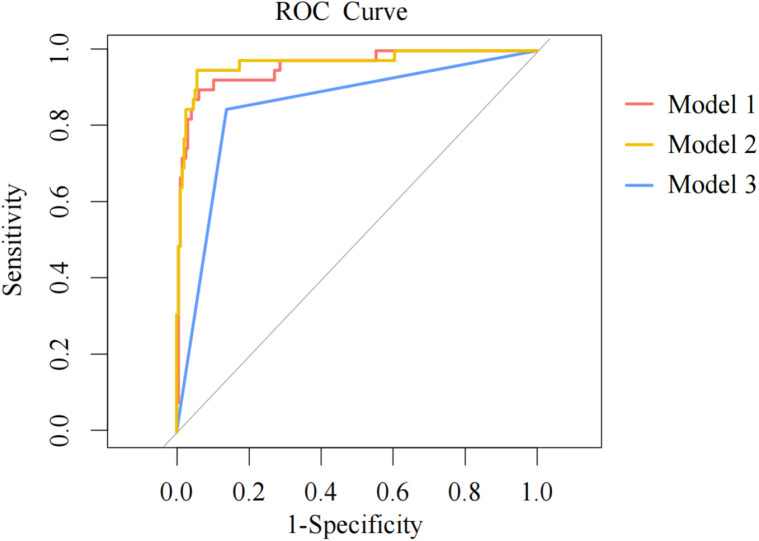
ROC curves for three models. ROC, receiver operator characteristics; AUC, area under the curve. The models are represented with curves in different colors. The dots on the curves are the optimal cutoffs. A larger AUC indicates that a model has greater capability to distinguish patients from non-patients and the prediction results are more accurate with better discrimination.

To validate the predictive model (Model 1), AUC of the three models’ ROC curves were calculated. Figure 3 demonstrates the ROC curves with the optimal cutoff values. Model 1 had the largest AUC, indicating that it had the best discrimination.

#### Construction of nomogram in training cohort

**Figure 4:**
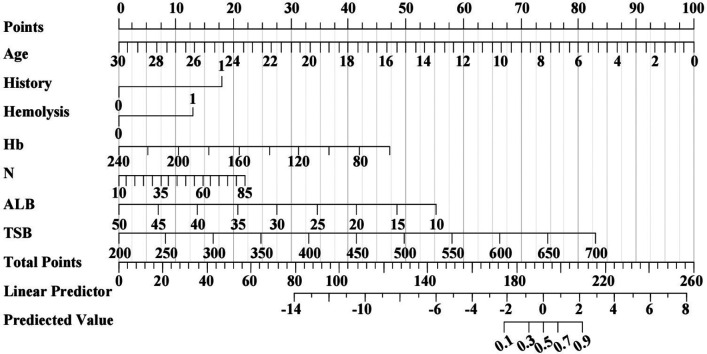
Prediction nomogram for neonatal phototherapy outcome created based on Model 1. The nomogram shows the weight of age, past medical history, presence of hemolysis, Hb, N%, ALB, and TSB in the training cohort. The line of each indicator is scaled with its range of values. The length of a line reflects the contribution of a factor to an outcome event. All scores are summed for a total point. The probability of prediction is resulted by drawing a perpendicular line at the total score.

In order to graphically represent the predictive value of each predictive variable for neonatal phototherapy outcome and provide a more specific explanation of the impact of each predictive variable on the prediction results, we constructed a nomogram based on Model 1, which included seven indicators: age, past medical history, presence of hemolysis, Hb, N%, ALB, and TSB. Scoring of each independent predictor was plotted into a straight line. The scores were summed and the total score was positioned on the bottom line to represent the probability of a predicted neonatal phototherapy outcome based on the admission data (Figure 4).

#### Calibration of Model 1

**Figure 5:**
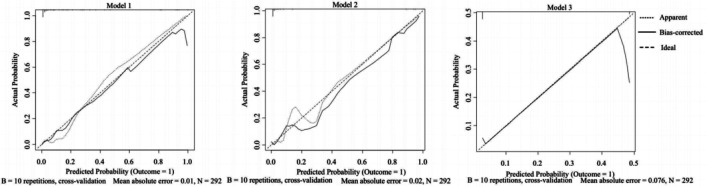
Calibration plots for three models: training cohort. The horizontal axis is the probability of a predicted phototherapy failure. The vertical axis is the probability of an actual event. The bias-corrected curve is the calibration curve after 10-fold cross-validation of a model. The 45° reference line represents the ideal calibration. The closer a solid line is to the reference line, the more probable the prediction is, showing better calibration of the model.

Figure 5 shows the calibration plots for the three models in the validation cohort. The bias-corrected curves are the calibration plots after 10-fold cross-validation. The 45° ideal curves were drawn as references. The bias-corrected curve of Model 1 was closer to the reference curve, suggesting that Model 1’s prediction would be closer to the probability of outcome.

#### Comparison of models

Table 7 shows the AUC, sensitivity, and specificity of each model with their 95% CI. AUC of Models 2 and 3 were compared with that of Model 1. The AUC difference was statistically significant between Model 3 and Model 1 (*p* < 0.017), which indicated that the new predictive model had greater power to predict phototherapy outcomes than the conventional criterion for phototherapy failure. Though Model 2 showed better sensitivity and specificity, its AUC was not significantly different from that of Model 1.

**Table 7:** AUC, sensitivity, and specificity of three models.

Model	AUC (95% CI)	Sensitivity (95% CI)	Specificity (95% CI)
1	0.958 (0.924–0.993)	0.897 (0.743–0.974)	0.937 (0.657–0.976)
2	0.969 (0.9364–1.000)	0.948 (0.769–1.000)	0.944 (0.379–0.980)
3	0.854 (0.793–0.915)	0.846 (0.618–0.926)	0.862 (0.476–0.903)

AUC, area under the curve; 95% CI, 95% confidence interval; Models 2 and 3 were respectively compared with Model 1. Bonferroni correction was used. *p* < 0.017 was statistically significant.

#### DCA

**Figure 6:**
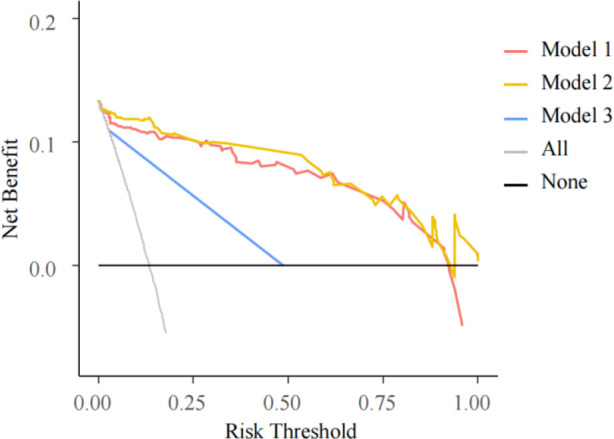
DCA for three models. The horizontal axis is the threshold probability. In the predictive model, when a patient’s probability of phototherapy failure reached the threshold, it was deemed that the phototherapy had failed and exchange transfusion therapy (ETT) was ordered. The vertical axis is the net benefit (NB), benefits of ETT after phototherapy failure minus the damage due to ETT despite successful phototherapy and the damage resulted from ETT not performed after phototherapy failure. The black solid line from 0.0 on the vertical axis represents 0 NB where phototherapies are successful for all patients, who require no ETT. The black oblique line is where ETT is required for every patient after all of their phototherapies fail. The curves are compared with the two reference lines of the extreme scenarios. The closer a curve is to the two reference lines, the less value the model has in application. A model is preferable when it is above the reference lines with a larger threshold range. NB is a backslash with a negative slope. A model is better with a greater NB under a given threshold.

DCA were performed on the three models (Figure 6). The decision curve of Model 3 was the closest to the reference lines, which suggested that it had no clinical predictive value. Compared with Models 2 and 3, Model 1 had a curve well above the reference lines with a large threshold range, which indicated that its predictive power could support clinical decision-making.

DCA evaluates the models by calculating their net benefits (NB). The DCA curves for both Models 1 and 2 were higher than that of Model 3, suggesting Models 1 and 2 to be more promising for clinical use.

#### NRI and IDI

NRI and IDI reflect changes in the difference between predictive probabilities. A greater IDI represents greater predictive power. Improvement is positive if IDI > 0 and negative if IDI < 0. IDI = 0 means that the new model fails to improve. It is similar for NRI. According to Table 8, when Model 2 was compared with Model 1, NRI and IDI were >0, indicating that Model 2 had greater predictive power. In contrast, when Model 3 was compared with Model 1, both NRI and IDI were <0, indicating that Model 3 had weaker predictive power than Model 1.

**Table 8:** NRI and IDI: Model 2 vs. Model 1, Model 3 vs. Model 1.

	NRI (categorical) (95% CI)	NRI (continuous) (95% CI)	IDI (95% CI)
Model 2 vs. Model 1	0.07700 (−0.019–0.173)	0.9813 (0.687–1.2755)	0.0762 (0.0283–0.124)
Model 3 vs. Model 1	−0.1006 (−0.2428–0.0415)	−0.9971 (−1.2909–0.7032)	−0.2817 (−0.3875–0.1758)

NRI, net reclassification improvement; IDI, integrated discrimination improvement; 95% CI, 95% confidence interval.

#### ROC curves

We used the optimal cutoffs from the ROC analysis in the training cohort as thresholds to divide the predictive probabilities in the validation cohort into a successful phototherapy and a phototherapy failure group, which was combined with the phototherapy outcomes (Table 9).

**Table 9:** Validation cohorts and prediction results based on different cutoffs, by hospital and combined.

Prediction value									
	Phototherapy outcome	Model 1		Model 2		Model 3		Total
	Success	Failure	Success	Failure	Success	Failure		
Actual value	Hospital 1	Success	25	1	26	0	24	2	26
		Failure	2	1	2	1	1	2	3
	Hospital 2	Success	7	0	7	0	7	0	7
		Failure	4	12	5	11	10	6	16
	Combined	Success	32	1	32	1	31	2	33
		Failure	4	15	5	14	11	8	19

Hospital 1, Children’s Hospital of Soochow University; Hospital 2, Suzhou Municipal Hospital.

#### AUC, sensitivity, specificity, and 95% CI

**Figure 7:**
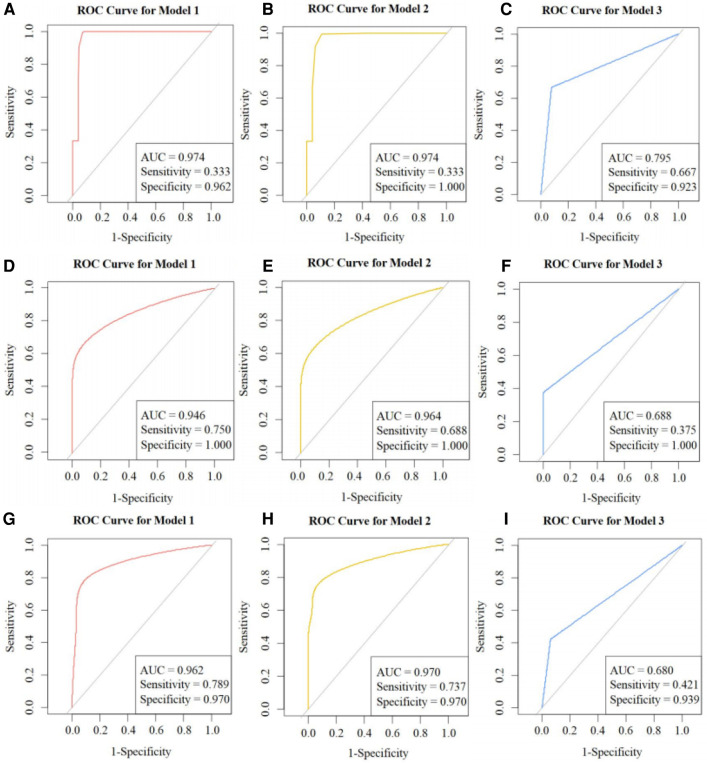
ROC curves: external validation. ROC, receiver operator characteristics; AUC, area under the curve. (**A**), (**B**), and (**C**) are the ROC curves and AUC of Models 1, 2, and 3 in the external validation cohort respectively, including their sensitivity and specificity calculated using the cutoffs from the training cohort. (**D**), (**E**), and (**F**) are the calibration plots of the three models in the external validation cohort. (**G**), (**H**), and (**I**) are the ROC curves of Models 1, 2, and 3 in the external validation cohort, including their sensitivity and specificity calculated using the threshold values.

Figure 7 shows ROC curves and calibration plots for our external validation. A, B, and C represent Models 1, 2, and 3, respectively, including their ROC curves and AUCs in the external validation cohort, and sensitivity and specificity calculated with the cutoffs from the training cohort. D, E, and F represent the calibration plots in the external validation cohort. G, H, and I represent the ROC curves for Models 1, 2, and 3 in the external validation cohort, showing the sensitivity and specificity calculated with the threshold values.

## Discussion

The permeability of the fetal BBB changes over time after birth and protects the brain from bilirubin. However, it remains immature in infants (17), who are therefore at greater risks for BE. In this study, we retrospectively collected data on multiple admission variables of infants with severe hyperbilirubinemia who received phototherapy. Some of the infants had poor phototherapy effects and were eventually given ETT. Some even developed BE. This suggests that routine phototherapy after admission may not benefit all infants with severe hyperbilirubinemia. After examining the admission variables, we constructed a predictive nomogram for phototherapy outcome using seven significant admission predictors, i.e., age, past medical history, presence of hemolysis, Hb, N%, ALB, and TSB levels. The predictive model was prospectively validated and showed good predictive power. The predictive nomogram using admission predictors may be a promising clinical instrument for early prediction of neonatal phototherapy outcome. Neonatologists may use the nomogram to determine if an infant should be considered for ETT and support their decision-making to initiate the therapy.

Age has been reported to be an important factor affecting TSB levels. In our study, the assignment value of age reached up to 100 within 24 h after birth in the nomogram, which suggests that age could be the most influential variable in the predictive model. The finding is consistent with a previous report in a Chinese population (18).

Though it was simplified as a Yes/No variable in the nomogram, past medical history of a neonate with severe hyperbilirubinemia can be complex, ranging from pre-eclampsia, hypertension, diabetes, virginal bleeding, mother and community culture and beliefs, breast conditions, reduced breastfeeding (19), pregnancy obesity (20) to abnormal labor (21). Other past medical history data such as mild infection (22), delayed umbilical cord ligation (23), and abnormal amniotic fluid and fetal movements could also be meaningful. Different prior conditions, such as premature birth, asphyxia, infection, hypothermia, hypoproteinemia, cranial hematoma, and hemolysis, could have impacted jaundice treatment and efficacy of phototherapy and resulted in inaccurate risk factor assessment and incomplete or even biased prediction.

Of the 292 enrolled newborns, 33 had ABO incompatibility, five had Rh disease, and one had G6PD deficiency. Hemolytic hyperbilirubinemia was found in the 39 newborns (13.4%), where phototherapy was successful in 23 newborns (59.0%). In contrast, success rate was considerably higher in those with non-hemolytic hyperbilirubinemia (91.3%, 231/253). According to a previous study, blood group incompatibilities may be linked with an higher incidence of BE (24). Presence of hemolysis was assigned as Yes/No in the monogram. Its lesser weight was attributable to the fact that ETT was given early in many Rh hemolytic patients, who were excluded from the current study. Hyperbilirubinemia is more likely in infants with G6PD deficiency, which occurs in 2.1% of male newborns (25). However, newborns are not routinely screened for G6PD deficiency in our hospital. The Hb levels in infants with ABO/Rh hemolysis are significantly lower in cases of severe hyperbilirubinemia (26). The criterion for severe neonatal anemia is an Hb between 60 and 90 g/L (11). As a result, Hb was assigned 40 in the nomogram, a rather low weight and similar to hemolysis.

Of the seven predictors included in our predictive model, N% was not statistically significant (*p* = 0.15). However, it is closely associated with infection, a known cause of neonatal hyperbilirubinemia (27). Phototherapy influences neutrophil/lymphocyte ratio by reducing cytokine and TSB levels (28). Umbilical bilirubin is a positive predictor for neonatal infections (29). Urinary tract infection is a possible reason for poor response to and increased duration of phototherapy in newborns with severe hyperbilirubinemia (30, 31). Given this evidence, we created the predictive model with stepAIC and included N% as a predictor despite its statistical insignificance and assigned it a low weight in the nomogram.

Risk factors for BE could be assessed using B/A ratio (32). A lower B/A ratio indicates that more bilirubin is bound with ALB while a higher B/A ratio reflects that there is more free bilirubin. BE is more likely to develop at high B/A ratios, as reported in the nervous system development in preterm neonates (33, 34). B/A ratio in conjunction with BIND score could increase sensitivity to 100% in predicting adverse outcomes of ABE (35). However, another cohort study revealed that both TSB and B/A ratio are strong predictors for neurotoxicity, but B/A ratio alone fails to improve prediction (36). A prospective random controlled multicenter study in the Netherlands found that compared with TSB alone, B/A ratio plus TSB does not improve neurodevelopmental outcomes in the management of hyperbilirubinemia in preterm neonates (37). As a result, the clinical value of TSB in predicting BE is more used whereas B/A ratio is not often calculated. We included ALB and TSB as predictors in our predictive model. The variance in ALB was not significant as shown in Table 1 (38.1, 36.4, 39.9) and its impact upon the predictive model was trivial (assignment < 20). In contrast, TSB was the most significant predictor for phototherapy outcomes, second only to age. As a known predictor for developing bilirubin neurotoxicity, TSB >20 mg/dl may lead to ABE (38). Bhutani et al. estimated the risk for chronic kernicterus to be 1/7 newborns with TSB >30 mg/dl (39).

Our nomogram is an easy-to-use instrument for personalized risk assessment with high accuracy (40). By quantifying the clinical indicators in the predictive model, we converted the scores into predictive probabilities in the nomogram to guide quick, improved clinical decision-making. A delay in initiating ETT could lead to serious clinical consequences. However, clinicians are often slowed to initiate clinically indicated ETT due to non-clinical impediments such as financial difficulties of the patient family and parents who are under- or misinformed about a procedure. The predictive model might expedite the decision-making process by only requiring the objective clinical data at admission.

## Limitations

The current study was performed at a single center with a limited sample size, although some infants in the validation cohort were from another hospital. Future studies should be performed to expand the work to multiple hospitals and increase sample size. Additionally, because most of the neonates included in the study were full-term, factors that can potentially influence phototherapy outcomes, such as body weight, gestational age, and use of probiotics, should be considered and investigated in the future.

## Conclusion

We developed and validated a model using seven predictors of clinical data at patient admission to predict phototherapy outcomes when treating newborns with severe hyperbilirubinemia. The new model can predict a phototherapy failure with improved accuracy earlier than using TSB levels alone. It may guide neonatologists to initiate ETT and other interventions earlier to minimize risks for serious complications of severe hyperbilirubinemia such as BE.

## Data Availability

The raw data supporting the conclusions of this article will be made available on request to the correspondence author.
